# Tribological Behavior of 3D-Printed Nanometer SiC and SiO_2_ Particle-Reinforced Polyamide 12 Composites by Selective Laser Sintering under Seawater Lubrication Condition

**DOI:** 10.3390/polym14194137

**Published:** 2022-10-03

**Authors:** Jingdong Ma, Guoyan Yu, Xianmin Wang, Jun Li, Jingquan Wu, Xianzhang Wang

**Affiliations:** 1 School of Mechanical Engineering, Guangdong Ocean University, Zhanjiang 524088, China; 2 Guangdong Provincial Marine Equipment and Manufacturing Engineering Technology Research Center, Zhanjiang 524088, China; 3 School of Chemistry and Environment, Guangdong Ocean University, Zhanjiang 524088, China

**Keywords:** polymers composite, seawater lubrication, selective laser sintering, friction, wear mechanism

## Abstract

Polymeric matrix composites have been widely used in the marine field. In this study, the tribological behavior under seawater-lubricated conditions of pure Polyamide 12 (PA12), micron-SiC and nanometer SiC and SiO_2_ particle-reinforced PA12 composites, which are prepared by selective laser sintering (SLS), were studied. The seawater absorption, hardness, contact angle and tribology performance were investigated. The results show that the addition of micron- and nano-SiC particles and nano-SiO_2_ particles could decrease the seawater adsorption and contact angle, and increase the hardness. Under seawater conditions, the addition of micro SiC particles can reduce the friction coefficient and wear loss, whereas the addition of nano-SiC and -SiO_2_ particles increases the corresponding values. The specimen printed with recycled powder has a higher friction coefficient, while having a better wear resistance. However, it increases the width and depth of the wear track in some locations. The wear mechanisms of the composite specimens are also analyzed. This was the result of the combined effects of fatigue wear and abrasive wear under seawater conditions. The latter plays a dominant role under seawater conditions. This study may provide a valuable reference for the further research and application of polymeric matrix composites in marine engineering equipment.

## 1. Introduction

In recent years, the friction and wear behavior of material under natural seawater-lubricated conditions, which has a wonderful environment, are widely investigated to obtain its lower friction coefficient and excellent wear resistance. Therefore, a number of researchers have carried out lots of studies on the characteristics of wear-resistant materials such as metals and alloys [1], polymers [2] and engineering ceramics [3,4,5] under natural seawater-lubricated conditions. In particular, polymers and polymeric matrix composites (PMCs) have excellent corrosion-resistant and tribological properties compared with metals and ceramics [6]. The polymers and PMCs test specimens are usually prepared by machining methods such as injection molding, hot-press molding technique, co-rotated parallel twin-screw extruder and wet hand lay-up process, etc. Compared with traditional machining methods, the additive manufacture (AM), known as 3D printing, has been widely applied over the last couple of decades. It has many advantages: geometrical complexity, toolless manufacturing and sustainable manufacturing [7]. Selective laser sintering (SLS), as an AM machining method, is a type of industrial powder bed fusion (PBF) process for the fabrication of polymeric components, which is widely used in the preparation of polymers and PMCs. In this study, a large number of test specimens were prepared by three consecutive prints to avoid material waste using SLS.

PA12 has good mechanical properties, low water absorption, a low relative density and melting point and low quality due to its chemical structure characteristic [8]. PA12 is one of the important materials used in the selective laser sintering process, and PA12 and PA12 matrix composites are the most common ones. Daniel B. Roppenecker [9] investigated the influence factors of friction coefficients for selective laser sintering, finding that the anisotropic material behavior, contact pressure, contact areas and intensive finishing treatment significantly affects the friction coefficient of the sample. Andreas [10] also studied the tribological anisotropy of selective laser-sintered PA12 components. The results show that the longest running-in-phase and lowest wear was found in the layer orientation parallel to the load direction, whereas a $45^{{}^{\circ}}\;\mathrm{and}\;90^{{}^{\circ}}$ orientation leads to a longer running-in-phase as higher wear at a $R_{z}$ 1.5 μm. Saleh [11] evaluated the three different orientations’ (X, Y, Z) effects on the tribological behaviors of laser-sintered PA12. The experimental results revealed that under dry conditions, the friction coefficient, and the specific wear rate of PA12 specimens oriented along X and Z axes, are significantly larger than when oriented along the Y-axis. In addition to the study of PA12 selective laser sintering samples, PA12 matrix composites selective laser sintering samples are also studied.

Wplyw et al. [12] added glass fiber into a PA12 matrix and prepared samples in different positioning directions on the construction platform. They found that the model direction positioning clearly influences the value of the coefficient of friction, and the highest wear occurs for samples at an angle of $45^{{}^{\circ}}$. Bai et al. [13] investigated the tribological and mechanical properties of the laser-sintered PA12/MoS_2_ and PA12. Results show that the coefficient of friction and wear rate of the laser-sintered PA12/MoS_2_ were reduced. Our previous study investigated the mechanical and tribological properties of 3D-printed PA12 and SiC/PA12 composite by SLS [14]. We found that the tribology behaviors of the SiC/PA12 composite were improved compared with the PA12 matrix. The study tribology properties under seawater lubrication of the PA12 and PA12 matrix composites are rare, but the tribology analysis of conventional manufactured polymer samples in seawater has been extensively studied.

Several polymers (poly-ether-ether-ketone (PEEK), polyphenylene sulfide (PPS), polytetrafluoroethylene (PTFE), polyimide (PI) and ultra-high molecular weight polyethylene (UHMWP)), and reinforced polymers with carbon fiber (CF), glass fiber (GF), carbon nanotube (CNT) and graphene oxide (GO), exhibited excellent tribological behaviors under natural seawater-lubricated conditions. PEEK has significant mechanical strength, a high melting temperature, chemical inertness, high toughness, easy processing and wear resistance [15]. Chen et al. [16] comparatively investigated the tribological behaviors under seawater lubrication of CF/PEEK composites prepared by the hot-press molding technique. They found that the incorporation of CF can greatly improve the wear resistance of PEEK under seawater lubrication, and the CF/PEEK has better tribological behavior than that under dry conditions and pure water lubrication. Chen et al. [17] also studied the synergetic effects of PEEK-based composites reinforced with lubricant additive and reinforcement additives including CF, GF and bronze powder prepared using a hot-press molding technique, and showed that PTFE and CF worked synergistically to enhance the wear resistance of the reinforced PEEK composite. Zhang et al. [18] investigated the comparative evaluation of friction and wear properties of CF/PEEK- and CF/PTFE/graphite-filled PEEK for seawater hydraulic piston pumps/motors. The results showed that CF/PTFE/graphite-filled PEEK had a lower friction coefficient and much better wear resistance than CF/PEEK. PPS has low viscosity and considerable fragility, and when mixed with reinforcement materials it exhibits good mechanical and thermal resistance, physical and chemical properties and also dimensional stability [19]. Xu et al. [20] reported the tribological behaviors under seawater lubrication of PPS filled with hydrolysable nanoparticles prepared by the hot-press molding technique. They found that the addition of BN or SiC nanoparticles significantly enhances the tribological performance of PPS. PTFE has a wide operating temperature range, high chemical resistance and excellent non-stick properties [21]. Wang et al. [22] comparatively investigated the friction and wear behavior of PTFE in air, distilled water, seawater and 3.5 wt.% NaCl solution. They found that the friction coefficient of PTFE in seawater was slightly lower than that in distilled water, but the wear rate was a little higher. PI has many extraordinary characteristics, i.e., good thermal stability, high mechanical strength, chemical inertness, self-lubrication and superior corrosion resistance. [23,24]. Chen et al. [25] systematically investigated the effects of several carbon series additions including graphite (Gr), CF and CNT on the microstructures and tribological behaviors of PI-based composites under seawater lubrication. They found that the incorporation of any filler improved the wear resistance of PI under seawater lubrication. However, it did not decrease the friction coefficient. Qi et al. [26] carried out the study on the tribological mechanism of short carbon fibers (SCF) and aramid particle (AP)-reinforced PI composites prepared by the hot-press molding technique under dry and seawater lubrication conditions. The result showed that the AP was more effective than SCF for enhancing the tribological performance under dry conditions. Min et al. [27] synthesized the PI/GO nanocomposite films by in situ polymerization, and the friction and wear properties under different lubrication conditions were comparatively investigated. The result showed that PI/GO composites exhibited better tribological behaviors under seawater-lubricated conditions than other conditions. UHMWPE has lots of advantages: high wear resistance, low friction, high impact strength, ultra-low water absorption and excellent chemical stability [28,29,30,31]. Wang et al. [32] studied the tribological behavior of UHMWPE under seawater lubrication compared with dry sliding, lubrication of pure water and 3.5 wt.%NaCl solution. It was found that the wear rate of UHMWPE under the seawater lubrication and NaCl solution was much larger than that under other conditions, and such a kind of wear closely related to the corrosion of the counterface can be reckoned as indirect corrosive wear.

Although many tribological investigations on polymers and PMCs prepared by traditional machining methods have been carried out, the friction and wear behaviors of PA 12 and PA12 matrix composites prepared by SLS have been hardly acquired. The reports on the tribological behaviors of nanomaterial-reinforced PA12 composite have not been investigated so far. Due to these facts, the micron/nanoscale SiC and nanoscale SiO_2_-reinforced PA12 composites were prepared by SLS. Their tribological behaviors under natural seawater-lubricated conditions were investigated in this study. The seawater absorption and hardness (Shore D) tests of composites were also performed. Moreover, the wear surface and wear mechanism were analyzed by SEM, and EDS are discussed in detail.

## 2. Materials and Methods

### 2.1. Material and Specimens

PA12 (FS 3300PA, density of 0.48 g/cm^3^) as the matrix composite was commercially obtained from Hunan Farsoon High-Technology Co., Ltd. (Changsha, China). The micron and nanoparticles of SiC were supplied by Metal Metallurgy Co., Ltd. (Guangzhou, China). The nanoparticle of SiO_2_ was provided by XFNANO Co., Ltd. (Nanjing, China). The 4 wt.% micron-SiC and 2.5 wt.% nano-SiC as reinforcing fillers were mechanically mixed with the PA12 powder in an electric blender at a velocity of 500 rpm for 3 h. The specimens were prepared by SLS equipment (Hunan Huashu High-Tech Co., Ltd., Changsha, China), and the sintering characteristics are listed in Table 1.

After the first print, the unused powder was collected and recycled for the next printing, and it was blended at a velocity of 500 rpm for 2 h. Then, the specimens were sintered under the same conditions. The 1 wt.% SiO_2_ was added to the surplus material left from the second print. When each print was finished, the composites were cooled to room temperature for 24 h. Four kinds of PA12 matrix composites were denoted as PA12-1, PA12-2, PA12-3 and PA12-4. Their specific parameters are listed in Table 2.

**Figure 1:**
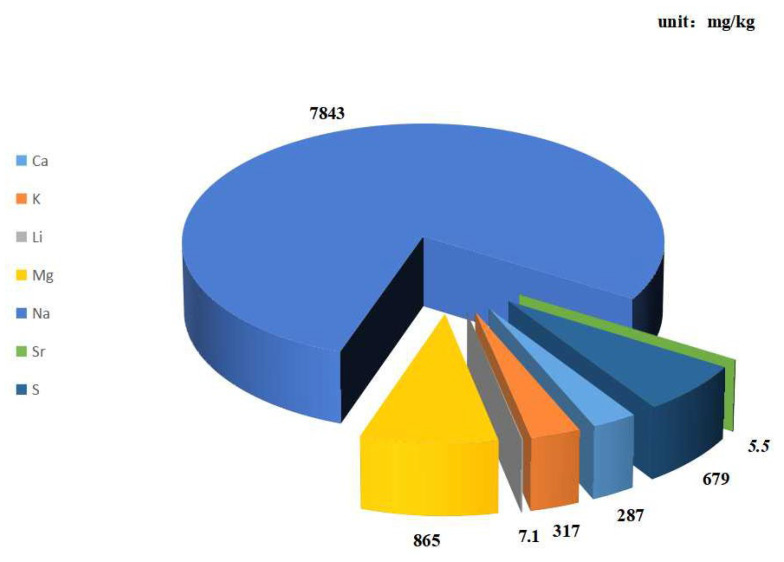
Chemical element of natural seawater.

The natural seawater, sourced from the Zhang Jiang sea area of the South China Sea, was used as a lubricant in the tribo-tests. The PH value of the seawater was 7.9 by the acidity meter (PH-100B, Lichen-BX instrument Technology, Shanghai, China). The chemical element content of seawater was measured according to the ICP-OES/MS (Perkin Elmer Optima 8000, Waltham, MA, USA), and the obtained values are shown in Figure 1.

### 2.2. Seawater Absorption

The seawater absorption tests of micron-nanometer SiC and nanometer SiO_2_-filled PA12 were performed according to ISO 62-2008 by immersing the five specimens in seawater for 24 h. The composites’ hardness were measured according to GB/T531.1-2008 on the shore-D scale, before and after immersion in seawater for 24 h. The seawater absorption can be obtained by

(1) $$\omega_{s}=\frac{m_{2}-m_{1}}{m_{1}}\times 100\%$$

where $m_{1}$ is the specimen mass and $m_{2}$ is the specimen mass after immersion.

### 2.3. Hardness Tests

The hardness of the PA12 and SiC/PA12 composites are measured on an LX-D pointer-type shore hardness tester at a load of 50 N with 10-s dwell times. Five points for each sample are measured to obtain an average value. Each hardness test was repeated five times, and the average value was obtained.

### 2.4. Friction and Wear Tests

The friction and wear tests of specimens under natural seawater-lubricated conditions were carried out on a universal mechanical tester (UMT-3, Bruker, Billerica, MA, USA) at a temperature of 25 °C in an air environment. The upper ball was 304 carbon steel with a diameter of 8 mm, and the bottom composite disks were made by SLS specimens with a size of 30 mm (length) × 30 mm (width) × 5 mm (height). The friction experiments were conducted on a normal load of 20 N, with rotation speeds of 90 rpm under dry conditions. The rotation diameter was 10 mm, and the duration was 20 min. The bottom composite specimens were completely immersed in seawater. Before the friction tests were performed, each specimen was immersed about for 24 h to calculate the wear loss accurately.

The friction coefficient was continually recorded by using a computer attached to the tester. After the friction tests, the wear loss of the specimens was calculated. To study the wear mechanism under dry and natural seawater-lubricated conditions of composite specimens, the microscopic surface morphology and elemental distribution of the wear tracks were measured by scanning electron microscope (SEM, MERLIN Compact, ZEISS, Jena, Germany) and energy-dispersive spectroscopy (EDS). Moreover, the contact angle of the composite specimens was measured by the contact angle meter (JY-82B Kruss DSA).

## 3. Results and Discussion

### 3.1. Seawater Absorption

**Figure 2:**
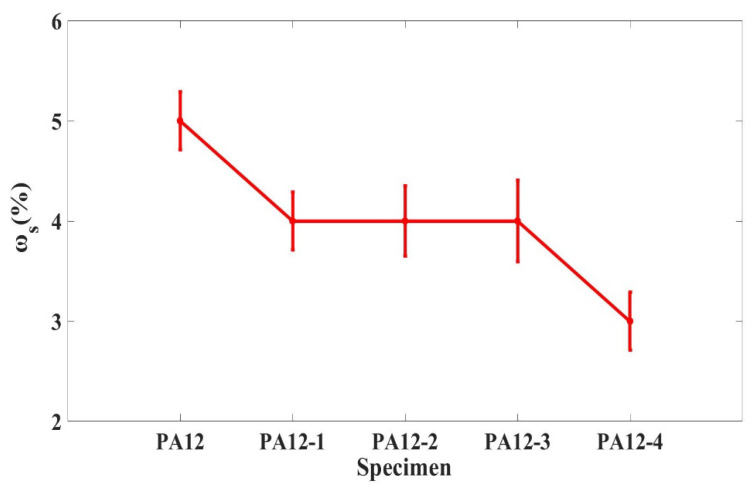
Seawater absorption of composite specimens.

Figure 2 shows the seawater absorption of composite specimens after immersing them for 24 h. It could be seen that the seawater absorption of the PA12 matrix composites was higher than that of the nanometer SiC and SiO_2_ particle-reinforced PA12 composites. The seawater absorption of the PA and PA12-1 were 5% and 4%, respectively. It demonstrates that adding macron SiC could decrease the seawater absorption of the composites. In contrast, the addition of micro with a lesser proportion and nanometer could reach the same effects. The printing of recycled powder does not affect the seawater absorption rate. The addition of nano-SiO_2_ can further decrease the adsorption to 3%; this might be because the particles filled the gaps in both the surface and bulk of composite specimens in the third printing progress. The seawater absorption of composites obviously decreased with the addition of micro and nanoparticles.

### 3.2. Hardness Tests

**Figure 3:**
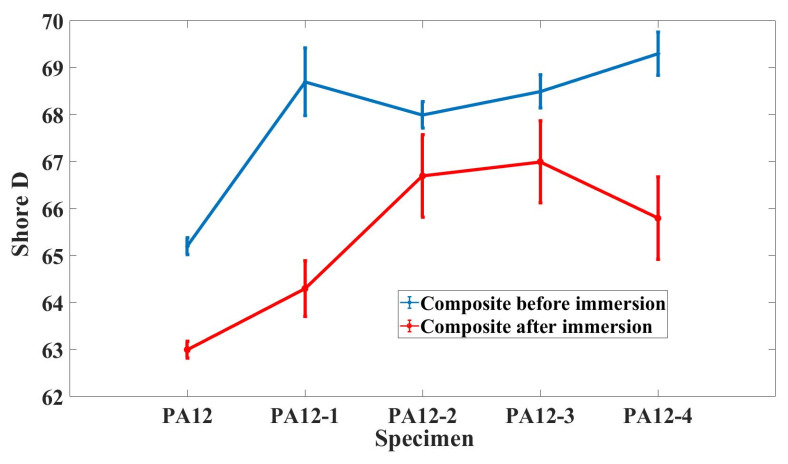
The hardness (Shore D) of composite specimens before and after immersion.

Figure 3 demonstrates the hardness (Shore D) change of composite specimens before and after immersion. As shown in Figure 3, the hardness (Shore D) of the PA12 was far below that of the filled PA12 composites (PA12-2, PA12-3, PA12-4). The hardness (Shore D) of the filled PA12 composite specimens increased greatly both before and after immersion. The addition of nanometer SiC makes the hardness of PA12-2 and PA12-3 decrease slightly under dry conditions. In the seawater environment, adding SiO_2_ nanoparticles could decrease the hardness of the composites. The change of hardness (Shore D) before and after immersion of the PA12-1 was relatively higher than in other cases.

### 3.3. Surface Characterization

**Figure 4:**
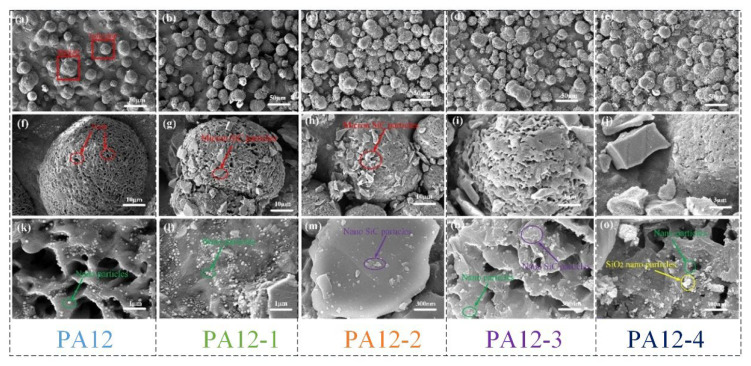
SEM images of surface with different magnifications for the PA composite specimens. (**a**–**e**) 500× magnification, (**f**–**j**) 1000× magnification, (**k**–**o**) 10,000× magnification.

The SEM helps observe the morphology of prepared composite specimens (PA12, PA12-1, PA12-2, PA12-3, PA12-4). Figure 4 shows the surface SEM images of the composite specimens with different magnifications. The numbers of near-hemispherical particles located on the surfaces of the PA12 and PA12-1 specimens, individually or stacked, which are clearly shown in Figure 4a–e, can be seen. The particle sizes of the hemispheres are relatively uniform, about 10–30 μm. Moreover, the particles emerged in an aggregation in local regions on the surface. The high magnification images are shown in Figure 4f–j. There are many microvoids on the surfaces. Compared with the PA12 surface, the hemispherical asperities of PA12-1, PA12-2, PA12-3 and PA12-4 are much rougher (see Figure 4g–j). The macro SiC can be seen on the surface of the unmelted powders. The images with larger magnifications are shown in Figure 4k–o, for more detailed structural information. In Figure 4k,i, there are many unknown nano spherical particles, which are a special material to improve the melting and forming properties of the powder. In Figure 4m,n, the nano-SiC and nanoparticles are spread over the micro SiC sheet or the voids. There are numerous nano-unknown and SiO_2_ particles on the surface of voids, as shown in Figure 4o. It should be noted that the SiC nanoparticles could not be seen near the apertures of voids, perhaps due to the particle shape and fluidity.

### 3.4. Contact Angle

**Figure 5:**
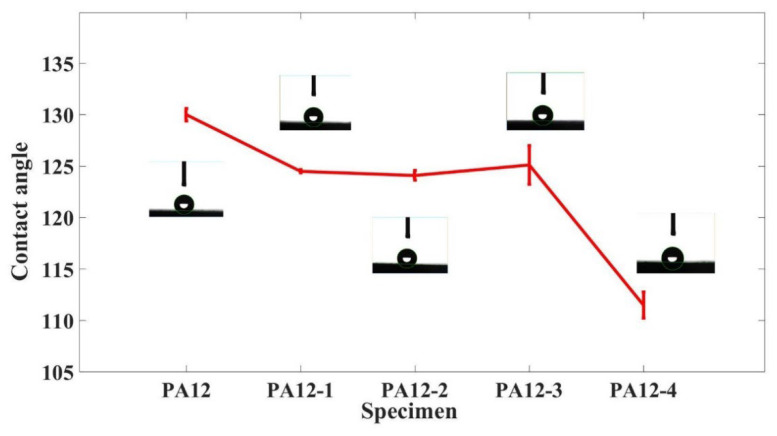
Contact angle of the composite specimens.

Figure 5 shows the contact angle of PA and its composite specimens (PA12-1, PA12-2, PA12-3, PA12-4) with seawater. The contact angle of composite specimens varies between 110 and 130 degrees, which indicates that they have hydrophobic properties. The contact angle of the PA12 matrix specimen is higher than that of the composite specimens. The contact angle of the composite decreases with the addition of micron- and nano-SiC particles, and further decreases greatly with the addition of nanometer SiO_2_. The main reason for this is that the addition of the micro and nanoparticle increases the degree of aggregation of particles and decreases the proportion of space on the surface. It is noted that the contact angle of PA12-2 and PA12-3 only has a slight change. 

### 3.5. Analysis of Friction and Wear

**Figure 6:**
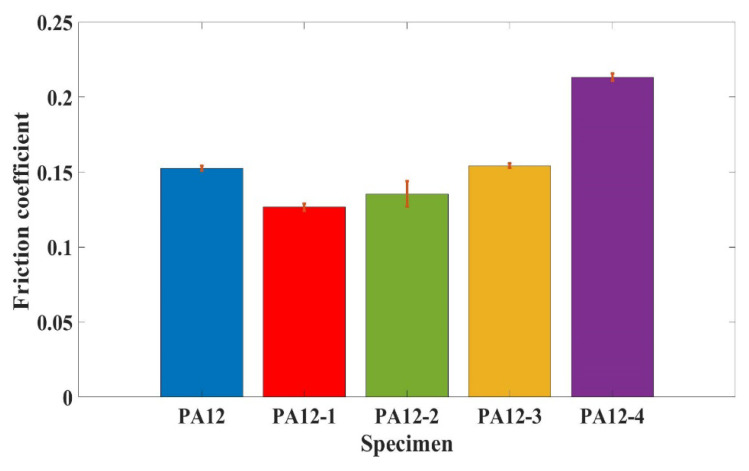
The friction coefficient of the specimens at a velocity of 90 rpm.

Figure 6 shows the average friction coefficient of composite specimens (PA12, PA12-1, PA12-2, PA12-3, PA12-4) under natural seawater-lubricated conditions. As can be seen from Figure 6, the friction coefficient under seawater lubrication is in the range of 0.13–0.23, which is relatively lower than that under dry conditions, due to the lubricating effect of seawater. The average friction coefficient of PA12-1 is the lowest among all the five cases. It is demonstrated that the addition of micron-SiC particles can decrease the friction coefficient. On the contrary, the addition of nano-size SiC and SiO_2_ increases the friction coefficient. Especially, the addition of nano-SiO_2_ increases the friction coefficient by about 30%. It is also founded that the friction coefficient of the printed specimen by recycled powder is a little higher than that of printed by pure PA12.

**Figure 7:**
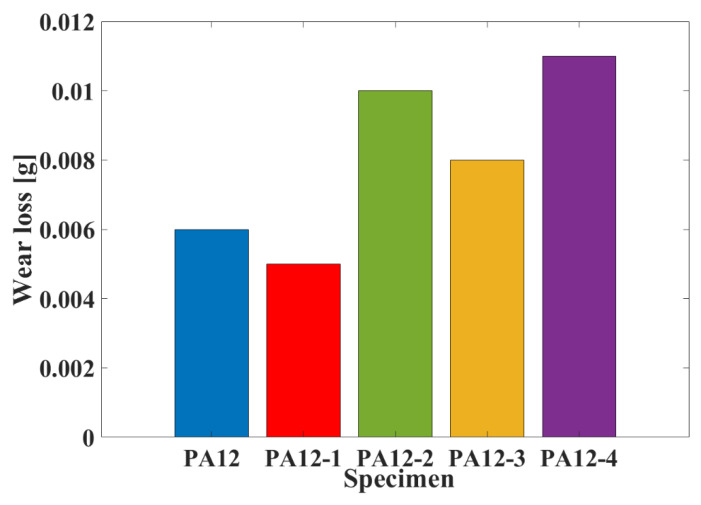
The wear loss of the specimens at a velocity of 90 rpm.

Figure 7 shows the wear loss of composite specimens (PA12, PA12-2, PA12-3, PA12-4) under natural seawater-lubricated conditions. As can be seen from Figure 7, the wear loss of specimen of PA12-1, with the addition of micron-SiC, was much lower than in the other cases. It illustrates that the specimen PA12-1 has the best wear resistance. The highest wear loss was obtained by the composite specimen of PA12-4. For the other cases, the specimens with a high friction coefficient have low wear loss. Compared with PA12-2, the wear loss of PA12-3 is lower. It suggests that the printed specimen by recycled powder has better wear resistance.

### 3.6. Analysis of Wear Surface and Wear Mechanism

**Figure 8:**
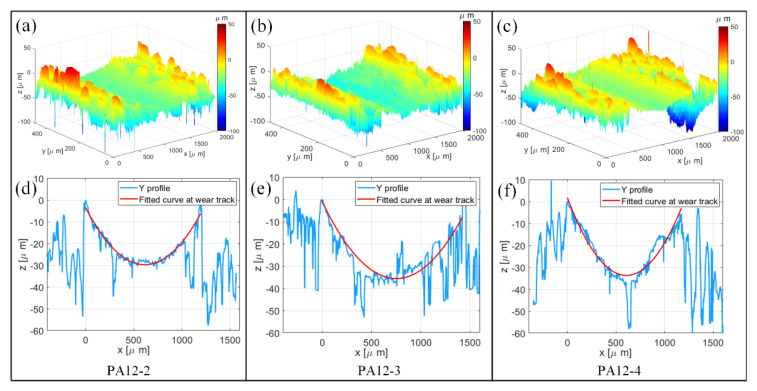
The worn surfaces of PA12-2, PA12-3 and PA12-4 specimens. (**a**–**c**) At 3D view and (**d**–**f**) Y profiles with fitted curves.

The 3D view and XY profiles of the worn surfaces at the wear tracks of PA12-2, PA12-3 and PA12-4, at a load of 20 N and a rotating velocity of 90 rpm, are shown in Figure 8. The 3D views of the figures are shown in Figure 8a–c. The Y profiles of the surface topographies are selected at wear tracks, as shown in Figure 8d–f. The Y profile shows the cross-section of wear track, then the curves at wear track of the three specimens are fitted. Then the width and depth of wear track can be obtained.

**Figure 9:**
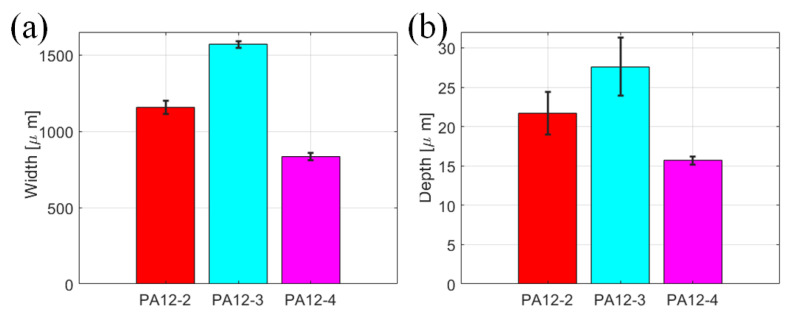
The width wear track dimensions of the PA12-2, PA12-3 and PA12-4 specimens. (**a**) Width, (**b**) Depth.

It can be seen from Figure 9 that the wear track of PA12-3 is wider than that of PA12-2 and PA12-4. This phenomenon can also be observed in the worn surfaces in a 3D view. The wear track width of PA12-3 is the largest among all the specimens. The specimen printed with recycled powder could increase the width and depth of the wear track, while the addition of nano-SiO_2_ particles could decrease the width and depth. The powder distribution on the surface also might impact friction and wear loss.

**Figure 10:**
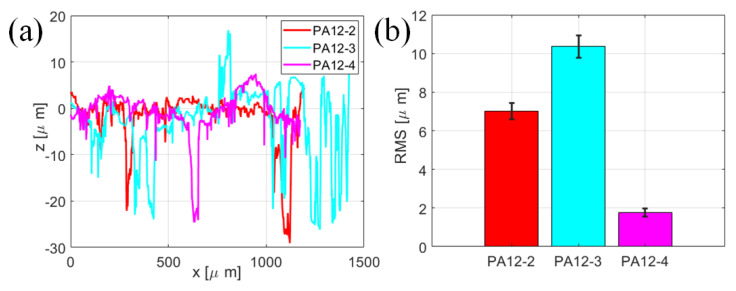
The analysis of selected profiles at the wear tracks of the PA12-2, PA12-3 and PA12-4 specimens. (**a**) Leveled profiles (**b**) RMS roughnesses.

The wear tracks are then leveled to make the surface flatten, i.e., the original measured height minus the fitted curve data. The leveled profiles of the specimens are shown in Figure 10a. One of the most important parameters that describes rough surfaces is the root mean square (RMS) height, which is calculated by the following equation:

(2) $$R_{q}=\sqrt{\frac{1}{N}\overset{N}{\underset{i=1}{\sum }}{\left(z_{i}-\bar{z}\right)}^{2}}$$

The profiles for the random y locations at wear tracks are chosen, and the RMS heights of the three specimens are presented in Figure 10b. As can be seen from Figure 10b, the roughness of the PA12-3 is larger than that of PA12-2. It demonstrates that the specimen printed by the recycled power has a rougher surface on the wear track. The roughness of PA12-4 is lower than that of PA12-3, implying that the addition of nano-SiO_2_ could make the wear track smoother and further improve the surface characteristics.

**Figure 11:**
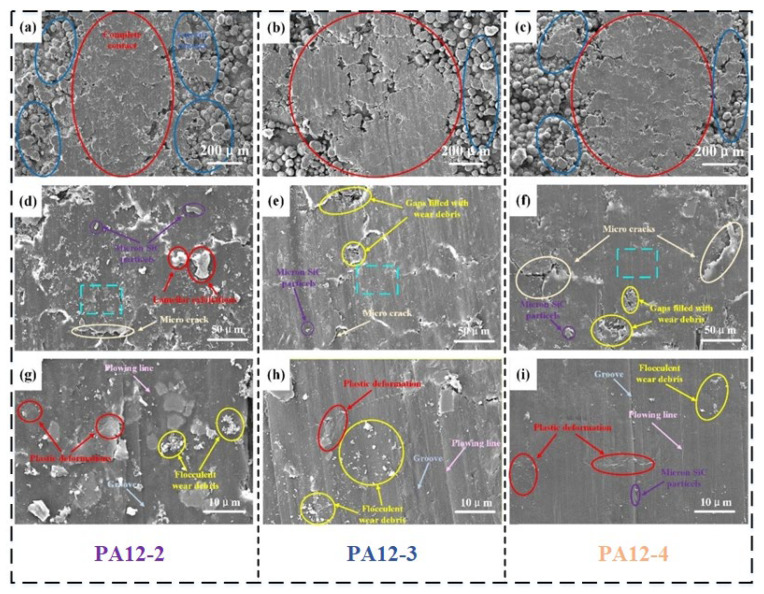
SEM images with various magnifications of wear surface under seawater lubrication. (**a**–**c**) 200× magnification, (**d**–**f**) 1,000× magnification, (**g**–**i**) 5,000× magnification.

The morphologies of the worn surfaces of PA12-2, PA12-3 and PA12-4 are shown in Figure 11. Due to the lubrication effect, the wear surface of composite specimens under seawater lubrication is smoother than in dry condition. Figure 11a–c shows the micro-structure inside and outside of the wear track of the PA12-2, PA12-3 and PA12-4 specimens. They show that the worn surface is characterized by serious plastic deformation and grooves, even micron-crack. In the central section of the wear tracks (in the red ellipse), the contact of asperities is almost complete. At the edges of wear tracks (in the blue ellipse), the plastic deformation of the asperities was produced during sliding. In Figure 11d–f, the micron-SiC particles are observed as well. The reinforced particle detachment was triggered due to the lower resistance of the polymer matrix to hold SiC particles. The micro-cracks can be clearly seen. The lamellar exfoliations are produced during sliding, which is one of the most distinctive characteristics of fatigue wear. Some micro-cracks can also be observed, some of which are the boundaries of deformed asperities. The narrow gap makes the structure on the surface compact. However, if the gaps between the asperities are wide, the detached wear debris and SiC particles are filled in. The lamellar exfoliation wear debris in the yellow ellipses resulted from fatigue wear. The high magnification images of the area in the bright blue blocks region are shown in Figure 11g–i. Lots of flocculent wear debris and serious plastic deformation were found on the surface of all the 3 specimens. The worn surface exhibits fatigue microcracks due to the loading cycles. In addition, the grooves and plowing lines can be seen clearly, which indicates that the effect of furrows is extremely obvious; one of the most distinctive characteristics of abrasive wear. PA12-3 specimen has more scratches than PA12-2 and PA12-4. This implies that the composite specimen is dominated by severe abrasive wear and fatigue wear. It might be convincing that the wear resulted from “plastic deformation crackle particle peeling”. When the shear stress is applied, the microcracks and wear debris gradually form during the sliding process. The wear surface exhibits significant plastic deformation. The three body wear results in smaller debris noticed on the worn surface. Therefore, the wear mechanisms of the composite specimens are the result of the combined effect of fatigue wear and abrasive wear.

**Figure 12:**
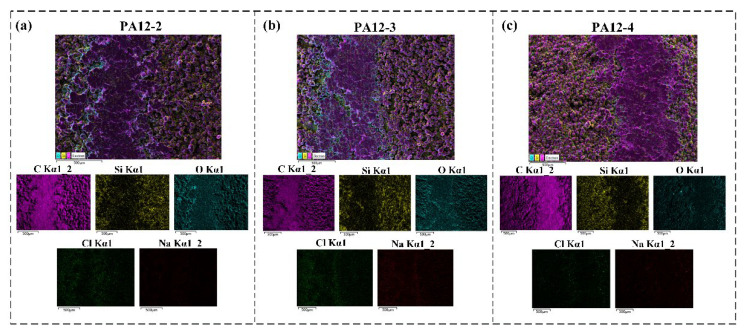
EDS layered elemental mapping images of worn surface of the composite specimen under sea water-lubricated conditions (**a**) PA12-2 (**b**) PA12-3 (**c**) PA12-4.

Figure 10 shows the elemental mapping of the worn surface under seawater-lubricated conditions. Figure 12a–c show the distributions of elements inside and outside the wear track of PA12-2, PA12-3 and PA12-4, respectively. The carbon (C), silicon (Si), oxygen (O), chlorine (Cl) and sodium (Na) on the inside and outside of the wear track of the composite specimens are measured by the mapping mode of EDS. Since the experiment is performed in the seawater environment, the elements shown in Figure 1 were considered. However, only a small amount of Cl and Na elements attached on the inside and outside of the wear tracks of the specimen were detected. It can be clearly seen that the distribution of carbon elements on the inside of wear track is relatively uniform, and the content of the locations—the valleys of the asperities on the outside of the wear tracks—are low. The distribution of silicon elements on the inside of the wear track is lower than that on the outside of wear track for all the specimens. This is because the SiC particles are worn off or washed out under seawater-lubricated conditions. The contents of the oxygen element on the edges of the wear track are significantly higher than that on the inside of the wear track, as shown in Figure 12c. An interesting phenomenon was found that the oxygen content of PA12-4 is lower than that of PA12-2 and PA12-3, while the silicon content is higher than other specimens. This demonstrated that the addition of nano-SiO_2_ could increase the silicon content of the wear track rather than the oxygen content. The underlying mechanism still needs further investigating.

## 4. Conclusions

In this study, the friction and wear properties of the nanometer SiC and SiO_2_ particle-reinforced PA12 composites that were fabricated by the SLS system under natural seawater-lubricated conditions were comparatively investigated. From the results, the following conclusions can be drawn:

(1) The addition of micron- and nano-SiC particles and nano-SiO_2_ particles could de-crease the sea water adsorption and contact angle, and increase the hardness for both dry and seawater condition.
(2) The addition of micron- and nano-SiC particles can reduce the friction coefficient and the specimen printed by recycled powder increase friction coefficient slightly, but has negligible difference compared with the PA12 specimen. The addition of nano-SiO_2_ increases the friction coefficient.
(3) The addition of micron- and nano-SiC particles and nano-SiO_2_ particles could increase the wear loss under seawater conditions. This might be because the reinforced particles were detached due to the lower resistance of the polymer matrix to hold them.
(4) The specimen printed by recycled powder could increase the width and depth of the wear track, while the addition of nano-SiO_2_ particles could decrease the width and depth.
(5) The wear mechanisms of the composite specimens are the result of the combined effect of fatigue wear and abrasive wear under seawater conditions. The latter plays a dominant role. The Silicon content of the inside of the wear track is much lower compared with the outside of wear track for all the specimens. This might be because some of the SiC particles are worn off or buried in the gaps of asperity boundaries.

## Figures and Tables

**Table 1:** The sintering characteristics.

Parameter	Preheated	Scanning Speed	Laser Power	Scanning Spacing
Value	170 [°C]	3000 [mm/s]	10 W	0.25 mm

**Table 2:** PA12-based composites.

Designation	Composition
PA12	Neat PA12
PA12-1	PA12 Gew. 10 wt % micron-SiC
PA12-2	PA12 Gew. 4 wt % micron-SiC + 2.5 wt.% nano-SiC
PA12-3	PA12 Gew. 4 wt.% micron-SiC + 2.5 wt.% nano-SiC (recycled powder)
PA12-4	PA12 Gew. 4 wt.% micron-SiC + 2.5 wt.% nano-SiC + 1 wt.% nano SiO_2_

## Data Availability

The data presented in this study are available on request from the corresponding author.
